# How to apply to a summer undergraduate research program

**DOI:** 10.1097/IJ9.0000000000000029

**Published:** 2017-05-11

**Authors:** Daniyal J. Jafree, Kiron Koshy

**Affiliations:** UCL Medical School, University College London, London, UK

**Keywords:** Medical student, Summer research, Undergraduate research, CV

## Abstract

Want to get more experience in research, but find it difficult as an undergraduate? A summer research project may be the answer and there are many ways to organize this. Various universities around the world offer summer undergraduate research programs. These tend to be very competitive; hundreds, even thousands of students can apply for only a handful of positions. However, with some proactivity and strong planning, diligent undergraduate students can get accepted onto these prestigious programs. In this article, we outline the steps involved in selecting and successfully applying to a summer undergraduate research program.

Various universities around the world offer summer undergraduate research programs (SURPs). These tend to be very competitive; hundreds, even thousands of students often apply for only a handful of positions. However, with some proactivity and strong planning, diligent undergraduate students can get accepted onto these prestigious programs.

## Why apply to a SURP and how do I find them?

Various institutions around the globe offer SURPs. These tend to be fully funded studentships exclusively for undergraduate students, featuring 8–12 weeks at a laboratory/clinical institute undertaking research under the supervision of one of the local research teams. Admission to these programs is extremely competitive and it is sensible to apply to many in 1 year to improve chances of success. If successful, these programs can skyrocket your experience in the research environment and are a fantastic boost for your curriculum vitae (CV).

SURP information is available throughout the Internet. Some webpages offer lists of SURPs such as that kindly produced by University of Cambridge’s Faculty of Biology^1^.

## Which SURPs should I apply to?

You should apply to the SURPs that are the most relevant to you. Each SURP tends to offer a number of different research projects that you can either select from or are assigned to. Most of these tend to be laboratory-focused, with a series of lectures, seminars, and tutorials to enhance your experience and maximize your output from the program.

### Before application

- **Check the admissions criteria.** Some SURPs only accept students from particular regions or countries, and some SURPs will only accept you if your latest end of year result is above a certain percentage. Some SURPs are designed for students with minimal previous research experience, while some are open to students from various research backgrounds. Typically, the SURP is applied for and carried out in the second year of an undergraduate science program, but these are variable.
- **Check the deadline.**
- **It is helpful to apply for a SURP that is relevant to your future career prospects**—it is great to be carrying out a SURP on the mechanisms of photosynthesis in plants but less so if you are aspiring to a career in academic neurosurgery. Particularly SURPs focus on specialized aspects, such as the Harvard Stem Cell Institute (HSCI) SURP, whereas some are more broadly focused on Biology, such as the Vienna Biocentre Summer Studentship. Despite this, research experience always provides generic skills, so it is still worth accepting a project in an area you will not later pursue.
- **Check the structure of the program**—some of these vary greatly. Some programs allow you to undertake the scheme at a date convenient for both you and the supervisor, whereas some are a fixed number of weeks.
- **Some programs will require you to contact supervisors in advance^2^.** It is advisable to contact supervisors at least 2 months in advance of the deadline for the SURP application.
- **Bear in mind that many of the SURP applications will require referees.** The requirements of referees vary from program to program. Some applications require a letter to be submitted directly to the program, some require a form to be filled out, and others will contact the referees after the deadline to directly obtain information about you. This should be taken into consideration when planning to complete your application before the deadline.

## What does the application require?

Application requirements vary depending on the SURP. Typically the application will consist of the following items:

- A cover letter or personal statement, stating why you are applying for the program, why you are suitable, and what you wish to obtain from the program. Some programs require several cover letters with a fixed word limit.
- Your CV.
- Your university academic transcript. This will be obtainable from your relevant university portal, and should include all your grades and examination scores to date.
- Material from referees (see above).

## Can I apply for multiple SURPs at 1 time?

Yes. It is highly recommended that you do, considering the extremely competitive nature of the SURPs. If you are lucky enough to get admission more than one, you must quickly make a decision and contact your second preference to inform them you have opted for another program. This will enable time for the admissions tutors to select the next candidate on their list.

## The outcome

Do not be disheartened if you get rejected from the SURPs of your choice. Most programs are extremely difficult to secure admission and any of the programs will be an excellent experience and adequate preparation for any research projects you are conducting in the coming years. If you are accepted, a welcome letter and an itinerary usually follow shortly after admission. Some of the programs have leisure and social activities included.

## Summary

- SURP programs are run at various institutions across the globe and are extremely competitive.
- There are various admission criteria and requirements that differ from program to program that must be considered before application.
- Successful admission requires a strong application, diligence, and patience.
